# The Lancet; or the Property and Privileges of Lecturers, Medical Societies, and Hospitals Considered

**Published:** 1824-03-01

**Authors:** 


### THE LANCET;

### OR THE

### *Property and Privileges of Lecturers, Hospitals, and Medical Societies, considered.*

——————Denique teipsum

Concute, num qua tibi vitiorum inseverit olim

Natura, aut etiam consuetudo mala.

This "minute instrument of mighty mischief" is in a great rage with us for having doubted the morality of publishing, *without authority or consent*, the lectures of a medical teacher. The Lancet professes to be utterly at a loss to understand such morality or immorality, comparing our squeamishness, on this point, to that of Beau Brommel, who held it to be immoral to drink porter, but virtuous to drink port wine with his cheese! Notwithstanding this very *apposite* comparison, we not only repeat our conviction that the transaction alluded to is immoral, (and illegal too, as the Lancet well knows,) but we have no hesitation in pronouncing it to be little less than *dishonest*—challenging the Lancet to offer any serious or just argument to the contrary. The only argument offered by the Lancet is this—that as parliamentary reports are permitted to be published, *though unauthorized*, so may the private prelections of a medical teacher. We are really astonished that any man pretending to a modicum of common sense, should attempt to draw a parallel between the public debates in a legislative assembly, where there is no property claimed, and the private lectures of an individual, constructed with great labour, and attended with great expence! Will not every man of common sense and common probity acknowledge, that the fee for admission to a lecture is merely a compensation for the personal benefit which the individual hearer may derive from the lecture so delivered, without the shadow of an implied right or permission for that hearer to publish the lecture next day for his own profit? If the Lancet can produce a single respectable testimony in favour of his parallel, or in negation of the view which we here take of the matter, we will consent to give up the argument.* Should such a monstrous principle, as that acted on by the Lancet, be once generally admitted and put in force, there would soon be an end to lectures entirely. Students would not be at the trouble or expence of feeing or attending lectures, if they could have them taken down and printed at sixpence per week. But granting that such an extreme case were not likely to occur, there is an evil, of great moment, which must inevitably attend this breach of confidence, of honour, and of honesty. We mean the perpetual restraint which it will place on the freedom of lecturers; preventing them from illustrating their precepts by examples drawn from public or private practice. What man will venture to relate the errors which he has committed or seen committed, when these are to be blazoned forth next day in the newspapers?†

Lectures are, from their very nature, unfit for the public eye—more especially the non-professional public, before whom they are now dragged and hawked about by unprincipled literary poachers. The most valuable portion of these lectures consists of the illustrations founded on occurrences in practice, and which are delivered in confidence. The system of espionage and forcible publication now pursued will, in a short time, annihilate this source of information for students, who will be the greatest sufferers in the end.

But it is not to the lecture-room alone that the baleful influence of a private spy and public informer is confined. His vampyre presence will chill the current of communication between preceptor and pupil in the clinical ward, and in the operating theatre. What surgeon's reputation is safe, with a man in mask at his elbow, taking notes for newspapers? What will be thought of the humanity, or rather inhumanity of the following disclosure? "A man was brought into the theatre (here the name of the hospital and operator is stated)

---

* The anecdote of Dougald Stewart (who may be considered as tolerable good authority on such a point) is well known. Observing a student one day taking notes at his lectures, he stopped suddenly, and declared he would not lecture any more while any one took notes. Such notes, he remarked, might be published, and such publication would be highly improper in itself, and injurious to the professor.

† It is to be particularly remembered, that the Lancet is not addressed exclusively to the profession, but to society at large; while its more striking parts are regularly forwarded to the metropolitan newspapers, in order to give greater publicity to the work, as we shall show farther on. Nay, it is posted up on boards in the streets, like Dr. Eady's advertisements, the Bonassus, or the murder of Weare! Let any one walk through the Strand, and convince himself.

to undergo the operation of lithotomy; *but after being on the table three quarters of an hour, five or six unsuccessful attempts to introduce the gorget into the bladder having been made, he was carried back to the ward.*" This statement was not less barbarous than *false.* The gorget *had* entered the bladder; but owing to the state of disease in the prostate gland and other unforeseen circumstances, the stone was so situated that no surgeon could have extracted it. And granting that the case had been as stated in the Lancet; of what use to the profession, we ask, was the publication of it? No man can command success in surgical operations—and if a surgeon fail from want of dexterity, he suffers mortification enough, Heaven knows, in the operation-room, without being put to the cruel and dæmoniacal torture of seeing the failure blazoned forth in the public prints. The man who could thus wantonly abuse the trust which is, by implied compact, vested in every student's honour, and thus lacerate the feelings of an individual, whose operations he was permitted to witness—

"Is fit for treason, stratagems, and spoils."

But this outrage on the common feelings and professional character of the medical officers of public institutions, must, should it continue, be attended with great detriment to those who seek refuge and relief in such asylums. The surgeon who has a secret spy upon his actions, ready to publish, and most likely to distort, every failure, will not risk his character by performing operations where there is much doubt of success—and thus many a wretched object will be left to pine in unmitigated sufferings, in consequence of this unprincipled system of dragging, *nolens volens*, every transaction that passes in an hospital before the public eye.

Rendered bold in injustice, from want of a curb, the Lancet commenced its predatory warfare on the literary property of the Medico-Chirurgical Society, unblushingly publishing the substance of the papers read at its meetings, and thus anticipating the volumes of their Transactions! The Society very properly put a stop to this iniquitous proceeding, and immediately all the scurrility of Billingsgate was let loose by the Lancet against the most respectable medical association in Europe! The arguments which the Lancet urges against this prohibition, on the part of the Society, are tragi-comic enough—namely, that the non-promulgation of the papers in the LANCET (where they would have "*a more immediate and extensive* REPUTATION!!") must be the destruction of *thousands of lives* annually!

"Now let us conceive that in this present month of January some facts of this description (useful facts) are presented to the Society, and accepted by it;—on the general application of which *the saving of thousands of lives, of millions of pangs and throes of agonizing humanity depends.* What would a stranger suppose that this Society, full, as it must be, of humanity and erudition, would do in such a case? He would imagine that he saw the members breaking forth with that tumultuous exultation—*quà data porta ruunt*—some at the front, some at the rear;—apothecaries and physicians, men-midwives and surgeons, dentists and oculists, licentiates and fellows, in most admired disorder, to publish the glad tidings far and wide, and pour balm into the wounds of human suffering. He certainly would be a little astonished, if, instead of this, he saw the society *deliberately contriving to conceal the knowledge it had acquired*, till the end of the year, *lest its profits* might be diminished in its account with the *bookseller*. Really Dr. Solomon was not to be *compared in meanness to this*. We would be glad to know what is the worst feature of *quackery*, but a profligate contempt of the welfare of mankind, when put in the balance against the pecuniary interests of the medical practitioner? And what can be a more complete specimen of this than the *deliberate suppression of useful knowledge?* If the guilt of this disregard of public welfare be in proportion to the smallness of the advantage which the society derives from it; *the wretch who adulterates drugs and destroys a patient, that he might pocket a penny on a pill, would not be entitled to rank so high in enormity as this Society, &c.*"—LANCET, SUNDAY, JAN. 11th.

We have copied the above as a fair specimen of the Lancet. The mock-heroic bombast, and sentimental lachrymation in the beginning of the passage, can only be equalled by the Billingsgate ribaldry, and the rancorous vituperation of its conclusion. The idea of the Transactions of the Medico-Chirurgical Society acquiring *reputation* by appearing in the LANCET, in company with the "Morbid Parts of Dr. Collyer," and other choice morceaux, is so exquisitely ludicrous that our sides were ready to burst on perusing the passage, which must afford no little entertainment to the members of that association.

The profession, and the public at large, must have remarked that, of late, scarcely a newspaper is published which does not contain extracts from certain weekly medical journals, and more especially the Lancet. Have the editors of newspapers all at once discovered a mine of information in these sources, which never before existed? No such thing. The manœuvre now is, to cull out extracts from these twopenny-halfpenny pamphlets, and send them to the newspapers the day before the pamphlets themselves are published, with a solicitation that they may be early inserted, or if that cannot be accomplished, they are paid for as *advertisements*, being considered the best kind of puff, and of the newest fashion. Now for an example. On Sunday the 11th of January, 1824, was published No. 2 of Vol. II. of the Lancet. On that day, of course, there being no shops open in the metropolis, the Lancet could be in the hands of very few—in fact, nobody scarcely sees the work on that day, unless he goes on purpose to Catharine Street, to purchase it. *At six o'clock* on the following morning (Monday) the tirade against the Medico-Chirurgical Society was published, as an extract from the Lancet, in that part of the *Morning Chronicle* which is notoriously set up and imposed, if not worked off, on the Saturday night preceding! Now we ask any man, who knows aught of the concerns of the press, whether it is likely that the editor of the Morning Chronicle spends his *Sundays* in searching for matter in medical journals *published that day*, to supply his newspaper, which is printed *that night, or rather the night before?* The fact of the extract being furnished by the Lancet itself—and that on the Saturday, is as clear as the sun at noon-day; —and practising such disingenuous arts as these, the Lancet has set itself up, not merely as a censor of printed works, but of the *conduct* of individuals in the medical profession! The *wall-bill* (like that of Eady or the Bonassus) for the Lancet of that week, was thus headed —"HUMBUG OF THE MEDICO-CHIRURGICAL SOCIETY." Such is the taste, such the decorum of a paper which is to regulate the moral and medical conduct of the profession! The following, among others, is a sample of its modesty. "We have the satisfaction of stating that persons who, from their situation, might be supposed to be hostile to the plan of publishing hospital cases, have acknowledged (where and when appears not on record) that *considerable talent* is displayed in the reports which we have presented to the profession." We acknowledge that "*considerable talent*" (especially of invention) is necessary to enable any one to traverse the wards of the metropolitan hospitals, *invisible* to human eyes, (which quality the Lancet lays claim to,) and, from the backs of tickets, make out a clear and authentic clinical history of the cases therein contained. This, we repeat, requires "considerable talent," especially when it is affirmed that such hospital reports must be far superior to those published by the hospital medical officers themselves! "*Our* reports (says the Lancet, with its usual *delicacy*) will have this advantage over any that might be published by the surgeons of the different institutions— that while *they* would have an interest in *concealing* many circumstances which might reflect discredit on themselves, &c." With this open insult and vile insinuation in its mouth, the Lancet has the effrontery to push itself, as a spy, into our hospitals; and there plunder and pilfer whatever comes in its way!

Considering the ways and means which the Lancet takes to degrade the profession and vilify its members, through the medium of the public prints, we think it is high time for the medical officers of hospitals to adopt precautions against the domiciliary visits of such lawless freebooters. The case of the Medico-Chirurgical Society is a sufficient example of the necessity of such measures.*

In our last number we hinted at the impropriety of jumbling politics, theatrical criticisms, and other non-professional subjects, in a medical review. We have always been of opinion that this is an unnatural union. Like the famous image in the vision of Daniel, it is formed of repulsive materials. The iron and clay will not coalesce—cannot long amalgamate.

——————————" Pergis pugnantia secum
" Frontibus adversis componere.'

Our rod of correction, though very gently applied, has had the desired effect in this respect. Since our last, the LANCET has drop-

---

* The Society of Apothecaries have also taken measures to secure their property against literary thieves. This has raised another howl against such old fashioned and squeamish morality.

ped, one by one, its theatricals, its politics, and even its chess problems. It has ceased, in some measure, to be ridiculous—but it steadily perseveres in being unjust. In its angry denunciation against us, it asserted, that it would not leave off its buffoonery, lest its pages should become "*as uniformly dull as those of the Medico-Chirurgical Review.*" To this we reply—better dull than indecent. The Medico-Chirurgical Review will never sully its pages by the details of unnatural crimes or propensities to excite the vulgar stare. Our Journal's olfactories are not, thank God, so apt as those of the Lancet at smelling out such dirty suits. It does not aim at the popularity of a pot-house or a smoaking-club. A time will come when the bubble of the Lancet's reputation will burst, blown up as it now is, by the baser passions of the baser portion of society. A time will come when the "Morbid Parts of Dr. Collyer" (as the Lancet's wall-bills used to be headed) will be too stale, even for the rabble—and when the hospital and lecture-room will be closed against professional traducers. It will then be seen whose pages are dullest.

We shall advert to but one more of the *just and honorable* callings taken up by the LANCET—*the censorship of individual speakers in medical societies*. This practice, with the Lancet's usual strength of argument, or rather its abortive sophistry, will be defended on the principle that newspapers are in the habit of criticising actors in a theatre, lawyers at the bar, and members of parliament in the senate. It will be of no use to urge that the members of a medical society associate for the purpose of communicating facts or discussing doctrines—and not for the display of oratory. Criticisms, therefore, on the language or style of speaking, in these societies, are as unjust as they are injurious. They can only encourage declamation, and repress the communication of useful knowledge.

The LANCET has taken our advice on more than one important point, abusing us all the time for having offered that advice—a sure proof that the truth of the admonition was felt, though not acknowledged. We shall, therefore, conclude this notice by recommending the LANCET to set seriously about the work of reformation. We advise it to leave off its habits of literary pilfering—to cease pushing its uninvited and unwelcome visage into societies, hospitals, and lecture-rooms with the express design of breaking the eighth commandment—to relinquish the office of medical jackall to the newspapers, and pander to the worst passions of human nature—to abstain from vituperating the profession, by addressing criticisms and reports to the non-professional public—to post not up its placards with those of Warren's blacking, thereby doing all that it can to tarnish the characrer of physic and surgery—lastly, to confine its soi-disant "considerable talent" to the legitimate objects and pursuits of other journals, the publication of honestly-acquired original papers, or the review of printed works. Whenever the Lancet restricts its operations within the rules of professional propriety and decorum, it will find most of its cotemporaries (now stigmatized as "cramped and debilitated by the trammels of professional sophistry,") its equals, and many of them its superiors.

